# Supplementary material for: Victimization status among persons with disabilities and its predictors: Evidence from Bangladesh National Survey on Persons with Disabilities
Source: PLoS One. 2025 Feb 24;20(2):e0304752. doi: 10.1371/journal.pone.0304752 (PMC11849822; doi:10.1371/journal.pone.0304752)
Supplement: S1 File — Supplementary Table 1: Age -specific distribution and association with victimization among persons with disabilities in Bangladesh. Supplementary Table 2: Factors associated with victimization status among persons with disabilities aged 0–17 years in Bangladesh. Supplementary Table 3: Factors associated with victimization status among persons with disabilities aged 18–59 years in Bangladesh. Supplementary Table 4: Factors associated with victimization status among persons with disabilities aged ≥60 years in Bangladesh. (DOCX) [file pone.0304752.s001.docx]

**Supplementary table 1: Age -specific distribution and association with victimization among persons with disabilities in Bangladesh.**

| **Categories (in years)** | **Frequency (%)** | **Prevalence** | **Odds ratio (95% CI)** | **p-value** |
| --- | --- | --- | --- | --- |
| 0-4 | 120 (2.80) | 37.60 | 1.00 |  |
| 5-9 | 256 (5.97) | 47.23 | 1.62 (0.94-2.77) | 0.080 |
| 10-14 | 312 (7.26) | 56.69 | 2.23 (1.33-3.73) | 0.002 |
| 15-19 | 334 (7.79) | 61.48 | 3.05 (1.82-5.11) | 0.000 |
| 20-24 | 275 (6.40) | 55.18 | 2.27 (1.34-3.85) | 0.002 |
| 25-29 | 248 (5.77) | 56.90 | 2.33 (1.36-3.98) | 0.002 |
| 30-34 | 239 (5.57) | 52.39 | 2.07 (1.21-3.53) | 0.008 |
| 35-39 | 273 (6.35) | 47.13 | 1.44 (0.85-2.44) | 0.170 |
| 40-44 | 206 (4.79) | 52.26 | 1.69 (0.98-2.92) | 0.061 |
| 45-49 | 251 (5.85) | 45.27 | 1.31 (0.77-2.23) | 0.321 |
| 50-54 | 268 (6.25) | 40.45 | 0.97 (0.57-166) | 0.918 |
| 55-59 | 299 (6.97) | 38.53 | 0.99 (0.59-1.68) | 0.982 |
| 60-64 | 332 (7.73) | 33.39 | 0.62 (0.37-1.05) | 0.074 |
| 65-69 | 310 (7.21) | 32.68 | 0.67 (0.40-1.14) | 0.141 |
| 70-74 | 226 (5.20) | 24.27 | 0.42 (0.24-0.75) | 0.003 |
| 75-79 | 123 (2.87) | 22.23 | 0.41 (0.21-0.80) | 0.009 |
| 80-84 | 100 (2.33) | 20.06 | 0.32 (0.15-0.66) | 0.002 |
| 85-89 | 54 (1.25) | 18.93 | 0.23 (0.09-0.58) | 0.002 |
| 90-95 | 67 (1.55) | 19.06 | 0.24 (0.10-0.56 | 0.001 |

*Note: unadjusted multilevel regression model used to identify association between victimization status and age.*

**Supplementary table 2: Factors associated with victimization status among persons with disabilities aged 0-17 years in Bangladesh.**

| **Characteristics** | **Model 1: Null model** | | **Model 2: Individual level factors** | | **Model 3: Individual and household level factors** | | **Model 4: Individual, household and community level factors** | |
| --- | --- | --- | --- | --- | --- | --- | --- | --- |
|  | aOR | **95% CI** | **aOR** | **95% CI** | **aOR** | **(95% CI)** | **aOR** | **95% CI** |
| **Individual level factor** |  |  |  |  |  |  |  |  |
| **Respondent’s age in years** |  |  | 1.06^**^ | 1.01-1.12 | 1.06^**^ | 1.01-1.12 | 1.07^**^ | 1.01-1.12 |
| **Gender** |  |  |  |  |  |  |  |  |
| Male (ref) |  |  | 1.0 | 1.0 | 1.0 | 1.0 | 1.0 | 1.0 |
| Female |  |  | 0.87 | 0.59-1.27 | 0.86 | 0.58-1.27 | 0.85 | 0.58-1.25 |
| **Respondent’s year of schooling** |  |  | 1.00 | 0.98-1.00 | 1.00 | 0.98-1.00 | 0.99 | 0.98-1.01 |
| **Respondent’s occupation** |  |  |  |  |  |  |  |  |
| Agriculture (ref) |  |  | Na | Na | Na | Na | Na | Na |
| Blue collar worker |  |  | Na | Na | Na | Na | Na | Na |
| Pink collar worker |  |  | Na | Na | Na | Na | Na | Na |
| White collar worker |  |  | Na | Na | Na | Na | Na | Na |
| Student |  |  | Na | Na | Na | Na | Na | Na |
| Housewives |  |  | Na | Na | Na | Na | Na | Na |
| Unable to work |  |  | Na | Na | Na | Na | Na | Na |
| Others |  |  | Na | Na | Na | Na | Na | Na |
| **Marital status** |  |  |  |  |  |  |  |  |
| Married (ref) |  |  | Na | Na | Na | Na | Na | Na |
| Unmarried |  |  | Na | Na | Na | Na | Na | Na |
| Widowed/Divorced/Separated |  |  | Na | Na | Na | Na | Na | Na |
| **Household level factor** |  |  |  |  |  |  |  |  |
| **Religion** |  |  |  |  |  |  |  |  |
| Muslim (ref) |  |  |  |  | 1.0 | 1.0 | 1.0 | 1.0 |
| Others |  |  |  |  | 1.05 | 0.48-2.30 | 1.12 | 0.53-2.38 |
| **Wealth quintile** |  |  |  |  |  |  |  |  |
| Poorest (ref) |  |  |  |  | 1.0 | 1.0 | 1.0 | 1.0 |
| Poorer |  |  |  |  | 0.98 | 0.57-1.69 | 0.88 | 0.52-1.51 |
| Middle |  |  |  |  | 0.71 | 0.40-1.28 | 0.64 | 0.36-1.15 |
| Richer |  |  |  |  | 1.02 | 0.55-1.89 | 0.78 | 0.42-1.46 |
| Richest |  |  |  |  | 0.67 | 0.35-1.31 | 0.42^**^ | 0.20-0.88 |
| **Community level factor** |  |  |  |  |  |  |  |  |
| **Place of residence** |  |  |  |  |  |  |  |  |
| Rural (ref) |  |  |  |  |  |  | 1.0 | 1.0 |
| Urban |  |  |  |  |  |  | 1.04 | 0.56-1.95 |
| **Region of residence** |  |  |  |  |  |  |  |  |
| Barishal (ref) |  |  |  |  |  |  | 1.0 | 1.0 |
| Chattogram |  |  |  |  |  |  | 0.88 | 0.34-2.23 |
| Dhaka |  |  |  |  |  |  | 1.95 | 0.77-4.97 |
| Khulna |  |  |  |  |  |  | 0.31^**^ | 0.12-0.85 |
| Mymensingh |  |  |  |  |  |  | 0.57 | 0.21-1.57 |
| Rajshahi |  |  |  |  |  |  | 0.63 | 0.24-1.61 |
| Rangpur |  |  |  |  |  |  | 0.36^**^ | 0.14-0.92 |
| Sylhet |  |  |  |  |  |  | 0.57 | 0.27-1.55 |
| **Model summary** |  |  |  |  |  |  |  |  |

| Intra-class correlation (ICC) | 0.38^***^ | 0.37^***^ | 0.39^***^ | 0.33^***^ |
| --- | --- | --- | --- | --- |
| Variance of the random intercept | 1.41 (1.05-1.89) ^***^ | 1.42 (1.02-1.98) ^***^ | 1.448 (1.03-2.02) ^***^ | 1.26 (0.86 -1.85) ^***^ |

**Supplementary table 3: Factors associated with victimization status among persons with disabilities aged 18-59 years in Bangladesh.**

| **Characteristics** | **Model 1: Null model** | | **Model 2: Individual level factors** | | **Model 3: Individual and household level factors** | | **Model 4: Individual, household and community level factors** | |
| --- | --- | --- | --- | --- | --- | --- | --- | --- |
|  |  |  | aOR | 95% CI | aOR | (95% CI) | aOR | 95% CI |
| **Individual level factor** |  |  |  |  |  |  |  |  |
| **Respondent’s age in years** |  |  | 0.98^***^ | 0.97-0.99 | 0.98^***^ | 0.97-0.99 | 0.98^***^ | 0.97-0.99 |
| **Gender** |  |  |  |  |  |  |  |  |
| Male (ref) |  |  | 1.0 | 1.0 | 1.0 | 1.0 | 1.0 | 1.0 |
| Female |  |  | 1.07 | 0.79-1.45 | 1.10 | 0.81-1.50 | 1.11 | 0.81-1.50 |
| **Respondent’s year of schooling** |  |  | 0.99 | 0.97-1.01 | 0.99 | 0.97-1.01 | 0.99 | 0.97-1.01 |
| **Respondent’s occupation** |  |  |  |  |  |  |  |  |
| Agriculture (ref) |  |  | 1.0 | 1.0 | 1.0 | 1.0 | 1.0 | 1.0 |
| Blue collar worker |  |  | 0.70 | 0.46-1.08 | 0.71 | 0.46-1.08 | 0.69 | 0.45-1.05 |
| Pink collar worker |  |  | 0.65 | 0.38-1.12 | 0.72 | 0.42-1.24 | 0.68 | 0.39-1.17 |
| White collar worker |  |  | 0.67 | 0.43-1.06 | 0.72 | 0.46-1.15 | 0.74 | 0.47-1.17 |
| Student |  |  | 0.35^***^ | 0.18-0.69 | 0.39^***^ | 0.19-0.76 | 0.42** | 0.21-0.82 |
| Housewives |  |  | 0.74 | 0.46-1.20 | 0.75 | 0.46-1.21 | 0.74 | 0.46-1.19 |
| Unable to work |  |  | 0.99 | 0.67-1.47 | 1.06 | 0.72-1.58 | 1.05 | 0.71-1.57 |
| Others |  |  | 0.99 | 0.63-1.57 | 1.09 | 0.69-1.72 | 1.08 | 0.68-1.71 |
| **Marital status** |  |  |  |  |  |  |  |  |
| Married (ref) |  |  | 1.0 | 1.0 | 1.0 | 1.0 | 1.0 | 1.0 |
| Unmarried |  |  | 2.29^***^ | 1.63-2.69 | 2.37^***^ | 1.69-3.32 | 2.24^***^ | 1.60-3.14 |
| Widowed/Divorced/Separated |  |  | 1.32 | 0.92-1.57 | 1.33 | 0.92-1.92 | 1.34 | 0.92-1.92 |
| **Household level factor** |  |  |  |  |  |  |  |  |
| **Religion** |  |  |  |  |  |  |  |  |
| Muslim (ref) |  |  |  |  | 1.0 | 1.0 | 1.0 | 1.0 |
| Others |  |  |  |  | 0.66^*^ | 0.45-1.00 | 0.68 | 0.46-1.01 |
| **Wealth quintile** |  |  |  |  |  |  |  |  |
| Poorest (ref) |  |  |  |  | 1.0 | 1.0 | 1.0 | 1.0 |
| Poorer |  |  |  |  | 0.88 | 0.64-1.20 | 0.86 | 0.63-1.17 |
| Middle |  |  |  |  | 0.90 | 0.66-1.25 | 0.88 | 0.64-1.22 |
| Richer |  |  |  |  | 0.77 | 0.54-1.09 | 0.74 | 0.52-1.06 |
| Richest |  |  |  |  | 0.48^***^ | 0.32-0.73 | 0.44^***^ | 0.28-0.69 |
| **Community level factor** |  |  |  |  |  |  |  |  |
| **Place of residence** |  |  |  |  |  |  |  |  |
| Rural (ref) |  |  |  |  |  |  | 1.0 | 1.0 |
| Urban |  |  |  |  |  |  | 0.84 | 0.56-1.24 |
| **Region of residence** |  |  |  |  |  |  |  |  |
| Barishal (ref) |  |  |  |  |  |  | 1.0 | 1.0 |
| Chattogram |  |  |  |  |  |  | 0.52^*^ | 0.29-0.95 |
| Dhaka |  |  |  |  |  |  | 1.05 | 0.58-1.91 |
| Khulna |  |  |  |  |  |  | 0.42^**^ | 0.23-0.95 |
| Mymensingh |  |  |  |  |  |  | 0.47^*^ | 0.24-0.95 |
| Rajshahi |  |  |  |  |  |  | 0.55 | 0.30-1.00 |
| Rangpur |  |  |  |  |  |  | 0.36^**^ | 0.20-0.67 |
| Sylhet |  |  |  |  |  |  | 0.34^**^ | 0.17-0.67 |
| **Model summary** |  |  |  |  |  |  |  |  |
| Intra-class correlation (ICC) | 0.32^***^ | | 0.34^***^ | | 0.33^***^ | | 0.30^***^ | |
| Variance of the random intercept | 1.25 (1.06-1.46) ^***^ | | 1.30 (1.11-1.53) ^***^ | | 1.27 (1.08-1.50) ^***^ | | 1.21 (1.02-1.43) ^***^ | |

**Supplementary table 4: Factors associated with victimization status among persons with disabilities aged ≥60 years in Bangladesh.**

| **Characteristics** | **Model 1: Null model** | | **Model 2: Individual level factors** | | **Model 3: Individual and household level factors** | | **Model 4: Individual, household and community level factors** | |
| --- | --- | --- | --- | --- | --- | --- | --- | --- |
|  |  |  | aOR | 95% CI | aOR | (95% CI) | aOR | 95% CI |
| **Individual level factor** |  |  |  |  |  |  |  |  |
| **Respondent’s age in years** |  |  | 0.97** | 0.95-0.99 | 0.97** | 0.95-0.99 | 0.97^**^ | 0.95-0.99 |
| **Gender** |  |  |  |  |  |  |  |  |
| Male (ref) |  |  | 1.0 | 1.0 | 1.0 | 1.0 | 1.0 | 1.0 |
| Female |  |  | 0.80 | 0.51-1.27 | 0.82 | 0.52-1.30 | 0.86 | 0.54-1.38 |
| **Respondent’s year of schooling** |  |  | 0.94* | 0.90-0.99 | 0.95* | 0.90-1.00 | 0.94^*^ | 0.89-1.00 |
| **Respondent’s occupation** |  |  |  |  |  |  |  |  |
| Agriculture (ref) |  |  | 1.0 | 1.0 | 1.0 | 1.0 | 1.0 | 1.0 |
| Blue collar worker |  |  | 4.01* | 1.17-13.52 | 3.85* | 1.15-12.96 | 3.28 | 0.97-11.02 |
| Pink collar worker |  |  | 1.09 | 0.40-2.99 | 1.12 | 0.41-3.06 | 1.15 | 0.42-3.15 |
| White collar worker |  |  | 1.16 | 0.54-2.46 | 1.17 | 0.55-1.91 | 1.12 | 0.52-2.38 |
| Student |  |  | - | - | ^-^ | - | - | - |
| Housewives |  |  | 0.83 | 0.35-1.96 | 0.81 | 0.34-1.91 | 0.69 | 0.29-1.62 |
| Unable to work |  |  | 0.92 | 0.52-1.63 | 0.93 | 0.53-1.66 | 0.95 | 0.53-1.69 |
| Others |  |  | 1.58 | 0.76-3.31 | 1.62 | 0.77-3.39 | 1.65 | 0.79-3.48 |
| **Marital status** |  |  |  |  |  |  |  |  |
| Married (ref) |  |  | 1.0 | 1.0 | 1.0 | 1.0 | 1.0 | 1.0 |
| Unmarried |  |  | 1.65 | 0.39-7.00 | 1.68 | 0.39-7.26 | 1.52 | 0.35-6.60 |
| Widowed/Divorced/Separated |  |  | 0.91 | 0.58-1.43 | 0.92 | 0.59-1.44 | 0.91 | 0.58-1.43 |
| **Household level factor** |  |  |  |  |  |  |  |  |
| **Religion** |  |  |  |  |  |  |  |  |
| Muslim (ref) |  |  |  |  | 1.0 | 1.0 | 1.0 | 1.0 |
| Others |  |  |  |  | 0.84 | 0.48-1.47 | 0.85 | 0.48-1.49 |
| **Wealth quintile** |  |  |  |  |  |  |  |  |
| Poorest (ref) |  |  |  |  | 1.0 | 1.0 | 1.0 | 1.0 |
| Poorer |  |  |  |  | 0.89 | 0.57-1.39 | 0.84 | 0.16-0.79 |
| Middle |  |  |  |  | 0.71 | 0.43-1.15 | 0.94 | 0.44-2.02 |
| Richer |  |  |  |  | 0.93 | 0.55-1.56 | 0.31 | 0.13-0.70 |
| Richest |  |  |  |  | 0.72 | 0.40-1.32 | 0.71 | 0.36-1.38 |
| **Community level factor** |  |  |  |  |  |  |  |  |
| **Place of residence** |  |  |  |  |  |  |  |  |
| Rural (ref) |  |  |  |  |  |  | 1.0 | 1.0 |
| Urban |  |  |  |  |  |  | 0.77 | 0.43-1.36 |
| **Region of residence** |  |  |  |  |  |  |  |  |
| Barishal (ref) |  |  |  |  |  |  | 1.0 | 1.0 |
| Chattogram |  |  |  |  |  |  | 0.35^*^ | 0.16-0.79 |
| Dhaka |  |  |  |  |  |  | 0.94 | 0.44-2.02 |
| Khulna |  |  |  |  |  |  | 0.31^**^ | 0.13-0.70 |
| Mymensingh |  |  |  |  |  |  | 0.14^***^ | 0.05-0.38 |
| Rajshahi |  |  |  |  |  |  | 0.46 | 0.21-1.01 |
| Rangpur |  |  |  |  |  |  | 0.35^*^ | 0.16-0.79 |
| Sylhet |  |  |  |  |  |  | 0.22** | 0.08-0.60 |
| **Model summary** |  |  |  |  |  |  |  |  |
| Intra-class correlation (ICC) | 0.33^***^ | | 0.33^***^ | | 0.33^***^ | | 0.30 ^***^ | |
| Variance of the random intercept | 1.27 (0.98-1.66) ^***^ | | 1.28 (0.98-168) | | 1.28 (0.97-1.68) ^***^ | | 1.20 (0.90-1.59) ^***^ | |
