# Supplementary material for: Victimization status among persons with disabilities and its predictors: Evidence from Bangladesh National Survey on Persons with Disabilities
Source: PLoS One. 2025 Feb 24;20(2):e0304752. doi: 10.1371/journal.pone.0304752 (PMC11849822; doi:10.1371/journal.pone.0304752)
Supplement: Attachment — Submitted filename: Reviewer_response_Author for Victimiization.docx [file pone.0304752.s005.docx]

**Authors’ response to reviewers’ comments**

| **Reviewer’s’ comment** | **Authors’ comment** |
| --- | --- |
| **Reviewer #1** |  |
| comments are attached  Abstract: The following two statements in the same abstract seems contradictory. “like Khulna and Rangpur was associated with lower likelihoods of being victimized” AND “residence in certain divisions such as Chattogram, Khulna, Mymensingh, Rangpur, and Sylhet reported higher likelihoods of victimization compared to those in the Barishal division”. | We have now revised the results section of the manuscript to address your comment. This now reads-  *The study found that 44% of persons with disabilities in Bangladesh experienced victimization, predominantly involving neighbours (90.64%), relatives (43.41%) friends (28.41%), and family members (27.07%). Among persons aged 0-17 years, increasing age was associated with higher likelihood of being victimized, while residing in the richest households or certain divisions like Khulna and Rangpur was associated with lower likelihoods. Conversely, among respondents aged 18-95 years, increasing age was associated with lower likelihood of being victimized. Unmarried respondents had increased likelihood of victimization compared to married individuals. In contrast, individuals from the highest wealth quintile, as well as those living in divisions like Chattogram, Khulna, Mymensingh, Rangpur, and Sylhet, had a lower probability of experiencing victimization compared to residents of the Barishal division.* |
| **Reviewer #2** |  |
| Title: The title is clear and concise; it reflects the focus of the study. | No change is required to address this comment. |
| Abstract: The abstract has done a great job summarizing this study, including background, methods, results, and conclusion. | No change is required to address this comment. |
| The writing of the manuscript is good; the logic in the flow from section to section is smooth. | No change is required to address this comment. |
| This study represents a valuable contribution to knowledge about victimization among persons with disabilities in Bangladesh. It carries useful insights that can inform policy and practice. This paper contributes to victimization among persons with disabilities in Bangladesh with key information in an area that is particularly under researched, especially in low- and middle-income countries. The study applied a sound methodology by using data from the 2021 National Survey on Persons with Disabilities and multilevel mixed-effect logistic regression analysis to ascertain the crucial predictors of victimization. | No change is required to address this comment. |
|  |  |
| Areas of Improvement Terms and Definitions: There are some terms that, through the manuscript, would have been better defined at the outset. While there was an attempt to explain the outcome variable, a more specific definition of what was meant by "victimization" and its forms, such as psychological, physical, and social, among others, would greatly aid in comprehension. An explanation for why the categories of harassment-for instance, based on ethnicity and religion-were combined into one dichotomous outcome variable would be better stated upfront and transparently. | We have now edited relevant sentences to provide the definition of victimization. It reads-  *Subsequently, we reclassified these responses into a single dichotomous variable for victimization status—whether individuals experienced discrimination or harassment based on their characteristics—categorized as "Yes" (indicating a positive response to any of the provided options) or "No" (reflecting negative or uncertain responses across all options).*  We considered harassment in general since ethnicity-related variables were not available in the survey. Although religion-related data was available, we found no significant differences in harassment based on religion. However, presenting these findings is beyond the scope of this study. |
| Discussion of Limitations: Even though the manuscript has stated that this very study is the first of its kind in Bangladesh, an elaboration on limitations was still expected. For example, self-reported victimization data might be subjected to recall bias or not be reported at all due to the possible stigma that might be associated with them. Specification about how this limitation may have influenced the results could help make this paper even stronger. | We have now revised the limitation section of the manuscript as follows to address your comment-  ………… *However, the primary limitations of this study include the analysis of cross-sectional data, which limits our ability to establish causality, as the findings are purely correlational. Additionally, the survey relied on self-reported victimization data, which may introduce the potential for misreporting certain experiences of violence or discrimination. Data were collected through questions posed to the respondents without validation opportunities, demonstrating the possibility of recall bias, although any such bias is likely to be random. Similarly, respondents were directly asked about their experience of any form of victimization, which may introduce the risk of underreporting or overreporting due to recall bias, stigma, or a preference for disclosing certain incidents while omitting others, particularly those that might contradict the experiences of their friends and family members. Moreover, aside from the factors adjusted in the model, health and environmental factors may contribute to discrimination against persons with disabilities, underscoring their importance for inclusion in the model. Unfortunately, these data were unavailable in the survey, limiting our ability to consider them. However, these limitations are unlikely to significantly impact the study findings given the analysis of large-scale data. Despite these limitations, the findings of this study will contribute to the development of national-level policies and programs.* |
| Ethical Considerations: Even though the manuscript maintains that no further ethical approval was necessary to undertake the study due to the de-identified nature of the data, it is desired that this section brings out considerations, if any, made to protect the privacy and rights of the respondents while data were being collected. | We have now revised the ethical approval section as follows to address your comment-  *The survey data we analyzed received ethical approval from the Ethics Committee of the Bangladesh Bureau of Statistics (BBS). Informed written consent was obtained from all participants or their legal guardians in cases where participants were minors (under 18 years old). We accessed the data from BBS by submitting a specific research proposal for this study. Before sharing the data, BBS de-identified it by removing all respondent-identifying variables. Since the data was de-identified, no further ethical approval was required.* |
| The focus on the victimization of persons with disabilities is so relevant since this population tends to be marginalized and underrepresented in research. These findings bear crucial implications for policy and strategies of intervention in Bangladesh and other similar contexts. | No change is required to address this general comment. |
| References Some references are not updated to reflect the most recent information, such as 2, 22, 31, 33, and 34. | We have now ensured references are UpToDate throughout the manuscript. |
