# Supplementary material for: Victimization status among persons with disabilities and its predictors: Evidence from Bangladesh National Survey on Persons with Disabilities
Source: PLoS One. 2025 Feb 24;20(2):e0304752. doi: 10.1371/journal.pone.0304752 (PMC11849822; doi:10.1371/journal.pone.0304752)
Supplement: Attachment — Submitted filename: Rahman et al PLOS One 2024 review 2.docx [file pone.0304752.s004.docx]

Thanks for your resubmission. However, the reviews are not addressed adequately. Please submit a revision letter and the changes in the manuscript with track changes.

Victimization Status Among Persons with Disabilities and its Predictors: Evidence from Bangladesh National Survey on Persons with Disabilities Dr Rahman et al.

The manuscript has merit to publish but its presentation is not up to the level.

Abstract:

The following two statements in the same abstract seems contradictory.

“certain divisions like Khulna and Rangpur was associated with lower likelihoods of being victimized” AND

“residence in certain divisions such as Chattogram, Khulna, Mymensingh, Rangpur, and Sylhet reported higher likelihoods of victimization compared to those in the Barishal division”.

Review 2: Please remove one or the other statement and re-write to make it clear.

Among 0-17 age bracket, increasing age was associated with higher likelihood and 18-95 age bracket, increasing age was associated with lower likelihood.  It seems the ages around 10-30 could have the highest prevalence of victimization. The current analyses did not show the more excellent inside of the data.  Understandably, very young children are less likely to be victimized, and so are older people. Adolescents and early to mid-adults are more victimized.  Find out the age range and the characteristics of people most victimized to propose any solutions for this.  The age group of 10 years could have a better reflection of the data.

Review 2: The above review still needs to be addressed. In the age group 0-17, the proportion is 51.83%, 18-59, 49.22%. From this data, it needs to be understood how victimization increases in 0-17 and decreases in 18 and above. Since the sample size is large, it is suggested that data be shown in more age group subdivisions to show where the pick is.

The rationale needs to be fully explained in the background. There are a few studies in Bangladesh. What did they do? What was the sample size? Where were the locations? What were the findings? These will lead you to the rationale for doing the current study. Please elaborate on references 23-26.

Soon, the authors reached Bangladesh. It is suggested that this is a global issue. Then, introduce Bangladesh with its population, problems, and hope for the future. Then, talk about disability and connect the problems and hopes for solutions.

Review 2: The above review is not addressed. Please submit the revision letter showing what changes were made by track changes.

Methodology:

Was it face-to-face data collection?

Review 2: It is not addressed.

"This subset of respondents was analyzed in alignment with the study's objectives". What are the study objectives? Are the authors referring to the main study objective? Please give a bit more context about the main study.

Review 2: First, data was collected from 155,025 participants with broad objectives. The current research is based on a sub-sample of 4,293 participants with disabilities. The objective of the current research is not presented.

At the end of the background, it seems the objective was “Therefore, we conducted this study to explore the extent of disability, as well as the factors associated with it, in Bangladesh”. However, the current sub-sample has addressed victimization and its associated factors.

It is suggested that the first study presents a broad objective, and then the current research presents a specific objective.

Please present all in a separate document as well as tract changes in the manuscript.

Do people understand the occupation categories?  I don't know well what blue-collar, pink-collar, and white-collar work occupations are. How the occupation data was collected?  What categories were in the questionnaire?  Please elaborate and explain. Bring these from the Table footnote.

Review 2: The above needs to be addressed.  It was suggested to bring the occupation categories such as Pink collar worker means [small business (capital up to taka 1000), business (capital over taka 10000), kabiraj /ojha/spiritual physician, village doctor and homeopathy doctor] shown in the footnote under the explanatory variables.

Have you considered an 80-year-old widow and a 20-year-old divorced woman to be in the same category? They should be in different groups.

Review 2: It has not been addressed. Have you tried if the widowed and divorced show difference? It should be answered.

This age cut-off is too broad and can't provide insight into the data.  An 18-year-old young adult and a 90-year-old adult should not be considered similarly.  It is suggested that the data be run for 0-5, 6-17, 18-59 (this is the most conservative minimum number of cut-off), 60+.  How many children are aged 0-1 years?  How do guardians know their levels of disability?  What was the age range of data collection then?  How was the consent collected for these children by BBS?  Please insert an ethics approval consent form in the appendix. I am unsure how these young children participated or how their consents were collected.

Review 2: The above is not addressed.

Table 1 title:  Please write "socio-demographic characteristics……………"

Why confidence intervals for Table 1?  These are not any inferential information, just baseline characteristics. I would suggest deleting the CIs.

Please report this footnote of Table 1 under methodology (variables).

Review 2: It is not addressed.

Table 2: It isn't easy to understand. What was the question?  For example, have you been victimized due to your sex or being male or female?

Also, present like below. Please re-do the Table 2.

| Characteristics | No at risk | Victimised, n (%) |
| --- | --- | --- |
| Male | 2000 | 50 (2.5) |
| Female | 3000 | 70 (2.7) |

Review 2: Not addressed. Currently, the data are not presented properly.

In Table 2, what the red marked row means? The objective is to understand victimisation among people with disability and the answer is almost 99% are victimized among people with disability if this is appropriate. Then what is the overall victimization 43.73% means? It seems averaging with age, gender. It is suggested to explain this findings.

Figure 1 with two decimal places is noise to read. Use no decimal places. Remove the “No bar” and write the number with different types of disabilities (autism, n=200), the bar will show only Yes column with 63%. The graph should have a vertical axis.

Review 2: it is not addressed.

Figure 2: Follow Figure 1. It has a vertical axis but the range is 120. So, poorly presented. Please use no decimal places and Yes bar only.

Table 3: Remove the “No” column, insert the number at risk. How many are under 0-17, 18-59. ………Without the numbers at risk, the Table is not completed.

Review 2: Not addressed. Please remove the NO column, add the number at risk and bring odds ratio (95% CI) from Table 5 to merge with this Table.

Table 4: How the ICC is related to the factors associated with victimisation? It just could be mentioned in the text only, based on the ICC what strategy was adopted. Please REMOVE Table 4.

Victimisation is similar in 0-17 and 18-59 years but much less in >60 years. However, later, the age group 18-59 and 60+ are merged which has diluted the results and has lost the findings.

(aOR: 0.97, 95% CI: 0.96-0.97); how the upper limit of the 95% CI is equal to the OR? It is expected to be 0.98.

Table 5: Please include the numbers at risk and the number (%) of cases throughout. For example,

|  | 0-17 |  | 18-59 |  | 60-95 |
| --- | --- | --- | --- | --- | --- |
| Male | 200 | 100 (50%) | 500 | 100 (20%) | 500 |
| Female | 300 | 100 (33%) | 500 | 150 (30%) | 500 |

The numbers are not presented anywhere. Good data but very poorly presented.
