# Supplementary material for: Victimization status among persons with disabilities and its predictors: Evidence from Bangladesh National Survey on Persons with Disabilities
Source: PLoS One. 2025 Feb 24;20(2):e0304752. doi: 10.1371/journal.pone.0304752 (PMC11849822; doi:10.1371/journal.pone.0304752)
Supplement: Attachment — Submitted filename: Point-by-point response to reviewerss comments.docx [file pone.0304752.s006.docx]

**Authors’ response to reviewers’ comments**

**Reviewer #1:** Major revision. Please submit a revision letter and the manuscript with track changes by addressing all the points raised.

Thanks for your resubmission. However, the reviews are not addressed adequately. Please submit a revision letter and the changes in the manuscript with track changes.

Victimization Status Among Persons with Disabilities and its Predictors: Evidence from Bangladesh National Survey on Persons with Disabilities Dr Rahman et al.

The manuscript has merit to publish but its presentation is not up to the level.

**Authors’ response:** Thank you very much for taking the time to carefully review our paper again. First, we apologize for overlooking your comments. Upon reviewing the journal’s decision email from the first round of revision, we found that no comments were attached in the PDF file sent by the journal, even though the email body indicated that your comments were attached. As a result, we only addressed the single comment in the email body, leaving the comments in the PDF file unaddressed. We regret this oversight and acknowledge that we could have reached out to the journal to inquire about the missing comments, but we missed that step. We apologize for this lapse. We have addressed all of your comments now and provided a point by point response below.

**Reviewer’s comments:**

Abstract:

The following two statements in the same abstract seems contradictory.

“certain divisions like Khulna and Rangpur was associated with lower likelihoods of being victimized” AND

“residence in certain divisions such as Chattogram, Khulna, Mymensingh, Rangpur, and Sylhet reported higher likelihoods of victimization compared to those in the Barishal division”.

**Authors’ response:** We have now rewritten the results section as follows-

The study found that 44% of persons with disabilities in Bangladesh experienced victimization, primarily involving neighbors (90.64%), relatives (43.41%), friends (28.41%), and family members (27.07%). Among persons aged 0-17 years, increasing age was associated with a higher likelihood of being victimized, while residing in the wealthiest households or in certain divisions like Khulna and Rangpur was associated with lower likelihoods. Conversely, among respondents aged 18-59 and ≥60 years, increasing age was associated with a lower likelihood of being victimized. Unmarried respondents aged 18-59 years had an increased likelihood of victimization compared to married individuals. For persons with disabilities aged ≥60 years, a higher level of schooling was associated with a reduced likelihood of being victimized. We also found divisional differences in the likelihood of victimization, with respondents residing in Chattogram, Khulna, Mymensingh, Rangpur, and Sylhet reporting lower likelihoods compared to those residing in the Barishal division, among persons with disabilities aged 18-59 and ≥60 years.

**Reviewer’s comments:**

Among 0-17 age bracket, increasing age was associated with higher likelihood and 18-95 age bracket, increasing age was associated with lower likelihood.  It seems the ages around 10-30 could have the highest prevalence of victimization. The current analyses did not show the more excellent inside of the data.  Understandably, very young children are less likely to be victimized, and so are older people. Adolescents and early to mid-adults are more victimized.  Find out the age range and the characteristics of people most victimized to propose any solutions for this.  The age group of 10 years could have a better reflection of the data.

Review 2: The above review still needs to be addressed. In the age group 0-17, the proportion is 51.83%, 18-59, 49.22%. From this data, it needs to be understood how victimization increases in 0-17 and decreases in 18 and above. Since the sample size is large, it is suggested that data be shown in more age group subdivisions to show where the pick is.

**Authors’ response:** We have thoroughly revised the manuscript and made the necessary changes. Following your direction, we created a 5-year age interval distribution and presented the association with victimization among this population in Supplementary Table 1 for better understanding. We hope this now clearly highlights where the peak prevalence occurs. Please take a look at Supplementary Table 1 below for your review.

| **Categories (in years)** | **Frequency (%)** | **Prevalence** | **Odds ratio (95% CI)** | **p-value** |
| --- | --- | --- | --- | --- |
| 0-4 | 120 (2.80) | 37.60 | 1.00 |  |
| 5-9 | 256 (5.97) | 47.23 | 1.62 (0.94-2.77) | 0.080 |
| 10-14 | 312 (7.26) | 56.69 | 2.23 (1.33-3.73) | 0.002 |
| 15-19 | 334 (7.79) | 61.48 | 3.05 (1.82-5.11) | 0.000 |
| 20-24 | 275 (6.40) | 55.18 | 2.27 (1.34-3.85) | 0.002 |
| 25-29 | 248 (5.77) | 56.90 | 2.33 (1.36-3.98) | 0.002 |
| 30-34 | 239 (5.57) | 52.39 | 2.07 (1.21-3.53) | 0.008 |
| 35-39 | 273 (6.35) | 47.13 | 1.44 (0.85-2.44) | 0.170 |
| 40-44 | 206 (4.79) | 52.26 | 1.69 (0.98-2.92) | 0.061 |
| 45-49 | 251 (5.85) | 45.27 | 1.31 (0.77-2.23) | 0.321 |
| 50-54 | 268 (6.25) | 40.45 | 0.97 (0.57-166) | 0.918 |
| 55-59 | 299 (6.97) | 38.53 | 0.99 (0.59-1.68) | 0.982 |
| 60-64 | 332 (7.73) | 33.39 | 0.62 (0.37-1.05) | 0.074 |
| 65-69 | 310 (7.21) | 32.68 | 0.67 (0.40-1.14) | 0.141 |
| 70-74 | 226 (5.20) | 24.27 | 0.42 (0.24-0.75) | 0.003 |
| 75-79 | 123 (2.87) | 22.23 | 0.41 (0.21-0.80) | 0.009 |
| 80-84 | 100 (2.33) | 20.06 | 0.32 (0.15-0.66) | 0.002 |
| 85-89 | 54 (1.25) | 18.93 | 0.23 (0.09-0.58) | 0.002 |
| 90-95 | 67 (1.55) | 19.06 | 0.24 (0.10-0.56 | 0.001 |

*Note: unadjusted multilevel regression model used to identify association between victimization status and age.*

**Reviewer’s comments:**

The rationale needs to be fully explained in the background. There are a few studies in Bangladesh. What did they do? What was the sample size? Where were the locations? What were the findings? These will lead you to the rationale for doing the current study. Please elaborate on references 23-26.

Soon, the authors reached Bangladesh. It is suggested that this is a global issue. Then, introduce Bangladesh with its population, problems, and hope for the future. Then, talk about disability and connect the problems and hopes for solutions.

Review 2: The above review is not addressed. Please submit the revision letter showing what changes were made by track changes.

**Authors’ response:** We have thoroughly revised the introduction section to address your comment. Specifically, we have provided more background on Bangladesh and critically reviewed the relevant literature. We would also like to mention that we did not find any relevant studies specific to Bangladesh, so the critical review was conducted using studies from other LMICs, in comparison with those from developed countries. Please see the introduction section of the revised manuscript.

**Reviewer’s comments:**

Methodology:

Was it face-to-face data collection?

Review 2: It is not addressed.

**Authors’ response:** We have now added the following sentences in the sampling strategy section of the methods to address your comment.

*Data were collected through face-to-face interview through structured questionnaire.*

Data were collected through face-to-face interview.

**Reviewer’s comments:**

"This subset of respondents was analyzed in alignment with the study's objectives". What are the study objectives? Are the authors referring to the main study objective? Please give a bit more context about the main study.

Review 2: First, data was collected from 155,025 participants with broad objectives. The current research is based on a sub-sample of 4,293 participants with disabilities. The objective of the current research is not presented.

At the end of the background, it seems the objective was “Therefore, we conducted this study to explore the extent of disability, as well as the factors associated with it, in Bangladesh”. However, the current sub-sample has addressed victimization and its associated factors.

It is suggested that the first study presents a broad objective, and then the current research presents a specific objective.

Please present all in a separate document as well as tract changes in the manuscript.

**Authors’ response:** The survey we analysed collected data from 155,025 respondents, of whom 4,293 self-reported a disability. We analysed this sample in alignment with the aims of this study, which has now been revised as follows at the end of the introduction section of the manuscript:

*Therefore, we conducted this study to explore the extent of victimization among persons with disabilities in Bangladesh, as well as the factors associated with it.*

Our revised analytical sample section now clearly presents this, and it reads:

*Of the survey sample, 4,293 respondents reported having a disability, comprising 2.79% of the total sample. This subset of respondents was analyzed in alignment with the study's objectives. The inclusion criteria utilized to derive this subset were as follows: (i) individuals self-reporting disabilities, and (ii) those who responded to questions concerning experiences of discrimination or harassment by individuals or groups on various grounds within 12 months preceding the survey.*

**Reviewer’s comments:**

Do people understand the occupation categories?  I don't know well what blue-collar, pink-collar, and white-collar work occupations are. How the occupation data was collected?  What categories were in the questionnaire?  Please elaborate and explain. Bring these from the Table footnote.

Review 2: The above needs to be addressed.  It was suggested to bring the occupation categories such as Pink collar worker means [small business (capital up to taka 1000), business (capital over taka 10000), kabiraj /ojha/spiritual physician, village doctor and homeopathy doctor] shown in the footnote under the explanatory variables.

**Authors’ response:** The occupation data was collected by directly asking respondents about their occupation, with a list of occupations provided for reporting, along with an open option to write in their occupation if it was not listed. We reclassified the reported occupations into the following categories: agriculture, blue-collar worker, pink-collar worker, students, housewives, unable to work, and others. The types of occupations included in each category are presented as a footnote to Table 1 in the revised version of the manuscript. It is important to note that this classification was necessary, as the survey data contained around 40 different types of occupations reported by respondents.

**Reviewer’s comments:**

Have you considered an 80-year-old widow and a 20-year-old divorced woman to be in the same category? They should be in different groups.

Review 2: It has not been addressed. Have you tried if the widowed and divorced show difference? It should be answered.

**Authors’ response:** Among persons with disabilities aged 0-95 years, we found that no respondents reported divorce, widowhood, or separation before the age of 20. Additionally, among persons aged 70 or older, there was no indication of divorce or separation, with only widowhood reported in this group. However, we categorized persons with disabilities aged 0-17 years as either unmarried or married, and those aged 70 or older as either married or widowed, respectively.

**Reviewer’s comments:**

This age cut-off is too broad and can't provide insight into the data.  An 18-year-old young adult and a 90-year-old adult should not be considered similarly.  It is suggested that the data be run for 0-5, 6-17, 18-59 (this is the most conservative minimum number of cut-off), 60+.  How many children are aged 0-1 years?  How do guardians know their levels of disability?  What was the age range of data collection then?  How was the consent collected for these children by BBS?  Please insert an ethics approval consent form in the appendix. I am unsure how these young children participated or how their consents were collected.

Review 2: The above is not addressed.

**Authors’ response:** Thank you for your insightful observation. We agree with your concern. However, we could not incorporate the adjustment as suggested due to the variation in the reporting of confounding variables across different groups. For instance, marital status and occupation data were largely missing for individuals aged 18 or below, as these questions were often inapplicable for younger participants, including those without disabilities. These variables, however, remain crucial for participants aged 18 and above. Consequently, aggregating data by five-year age groups and including all variables would lead to the loss of around half of the total sample in the regression model due to missing data on marital status or occupation. For a more comprehensive understanding, please refer to our regression results.

In response to your comment, we have now included a separate regression model stratified by age, using victimization as the dependent variable and age as the independent variable, without considering confounding variables. Results are provided in the supplementary file 1, with additional discussion for clarity. Please see our response to your earlier comments where we have pasted supplementary table 1.

In response to your comments on consent procedure, we would like to mention we have presented this information in the ethics approval section of the manuscript. This now reads-

*The survey data we analysed received ethical approval from the Ethics Committee of the Bangladesh Bureau of Statistics (BBS). Informed written consent was obtained from all participants or their legal guardians in cases where participants were minors (under 18 years old). We accessed the data from BBS by submitting a specific research proposal for this study. Before sharing the data, BBS de-identified it by removing all respondent-identifying variables. Since the data was de-identified, no further ethical approval was required.*

**Reviewer’s comments:**

Table 1 title:  Please write "socio-demographic characteristics……………"

Why confidence intervals for Table 1?  These are not any inferential information, just baseline characteristics. I would suggest deleting the CIs.

Please report this footnote of Table 1 under methodology (variables).

Review 2: It is not addressed.

**Authors’ response:** We have revised the table 1 title as suggested. It now reads-

*Table 1: Socio-demographic characteristics of the respondent (N=4,293)*

We have removed the 95% CI from Table 1. However, we have kept the table footnote as it was, as moving it to the methods section would make that section too cluttered. Additionally, this would make it more difficult for readers to understand the table, as they would need to refer to both the methods section and the table simultaneously.

**Reviewer’s comments:**

Table 2: It isn't easy to understand. What was the question?  For example, have you been victimized due to your sex or being male or female?

Also, present like below. Please re-do the Table 2.

| Characteristics | No at risk | Victimised, n (%) |
| --- | --- | --- |
| Male | 2000 | 50 (2.5) |
| Female | 3000 | 70 (2.7) |

Review 2: Not addressed. Currently, the data are not presented properly.

**Authors’ response:** We have revised the table according to your comments. Please see the updated version in the manuscript.

The question—“In the past 12 months, have you personally felt discriminated against or harassed on the basis of the following grounds?”—was framed to determine whether respondents felt victimized due to factors such as sex, age, or religion. However, segregated data, as exemplified in your suggested table, were not collected; therefore, we could not report it in that format.

**Reviewer’s comments:**

In Table 2, what the red marked row means? The objective is to understand victimisation among people with disability and the answer is almost 99% are victimized among people with disability if this is appropriate. Then what is the overall victimization 43.73% means? It seems averaging with age, gender. It is suggested to explain this findings.

**Authors’ response:** As mentioned in the outcome variable section of the methods that respondents were asked "In the past 12 months, have you personally felt discriminated against or harassed based on the following grounds?" They were presented with multiple options, including disability, ethnicity/immigration, sex, age, religion/belief, and other reasons. We presented this estimate in Table 2. Regarding disability one that you marked, this reported percentage of persons with disability reported disability as reasons for their victimization.

We have revised the table and the updated the results as per your comments. Please see the revised version of the manuscript.

**Reviewer’s comments:**

Figure 1 with two decimal places is noise to read. Use no decimal places. Remove the “No bar” and write the number with different types of disabilities (autism, n=200), the bar will show only Yes column with 63%. The graph should have a vertical axis.

Review 2: it is not addressed.

**Authors’ response:** We have revised the figure as per your comment. Please see the revised version of the manuscript.

**Reviewer’s comments:**

Figure 2: Follow Figure 1. It has a vertical axis but the range is 120. So, poorly presented. Please use no decimal places and Yes bar only.

**Authors’ response:** We have revised the figure as per your comment. Please see the revised version of the manuscript.

**Reviewer’s comments:**

Table 3: Remove the “No” column, insert the number at risk. How many are under 0-17, 18-59. ………Without the numbers at risk, the Table is not completed.

Review 2: Not addressed. Please remove the NO column, add the number at risk and bring odds ratio (95% CI) from Table 5 to merge with this Table.

**Authors’ response:** We have revised the table 3 as per your comment. Please see the revised version of the manuscript.

**Reviewer’s comments:**

Table 4: How the ICC is related to the factors associated with victimisation? It just could be mentioned in the text only, based on the ICC what strategy was adopted. Please REMOVE Table 4.

**Authors’ response:** We have deleted table 4 and revised relevant sentences to address your comment. Please see the revised version of the manuscript.

**Reviewer’s comments:**

Victimisation is similar in 0-17 and 18-59 years but much less in >60 years. However, later, the age group 18-59 and 60+ are merged which has diluted the results and has lost the findings.

(aOR: 0.97, 95% CI: 0.96-0.97); how the upper limit of the 95% CI is equal to the OR? It is expected to be 0.98.

Table 5: Please include the numbers at risk and the number (%) of cases throughout. For example,

|  | 0-17 |  | 18-59 |  | 60-95 |
| --- | --- | --- | --- | --- | --- |
| Male | 200 | 100 (50%) | 500 | 100 (20%) | 500 |
| Female | 300 | 100 (33%) | 500 | 150 (30%) | 500 |

The numbers are not presented anywhere. Good data but very poorly presented.

**Authors’ response:** We have revised the table as per your suggestion. We made three separate model for age 0-17, 18-59, and 60 and more years and explored their predictors. Please see the revised version of the manuscript.

Reviewer #2:

**Reviewer’s comments:**

Technical Soundness and Data Support:

The research is technically sound and appropriately designed for the question it addresses. The use of multilevel mixed-effect logistic regression is suitable, and the statistical analysis has been rigorously performed. However, improving the clarity of data presentation, particularly in the tables, would strengthen the argument. The conclusions are generally supported by the data, but more detailed consideration of the sample size and limitations is recommended.

**Authors’ response:** We have improved the data presentation by editing the table and figure in response to the first reviewer’s comment. Additionally, we have updated the limitations section to align with the current revised version of the manuscript.

**Reviewer’s comments:**

Statistical Analysis: The statistical methods were appropriately chosen and executed. Ensure that any assumptions made during the analysis are clearly described in the methods section for transparency.

**Authors’ response:** We have already presented the model assumptions and justification for using the multilevel logistic regression model in the statistical analysis section of the methods. Please see the revised version of the manuscript.

**Reviewer’s comments:**

Data Availability:

The current manuscript does not meet PLOS ONE’s data availability requirements. It is important to make the data underlying the findings fully accessible or to provide a valid justification if this is not possible. Please update the Data Availability Statement to comply with the journal's policy.

**Authors’ response:** We have updated the data availability statement as follows to address your comment:

*We analyzed secondary data available at the Bangladesh Bureau of Statistics (BBS) after submitting a research proposal. While we are not authorized to share the data file directly, we have ensured all relevant data for replication are included in the tables. Interested researchers can access the raw data file by submitting a research proposal to the BBS through the following link: https://bbs.gov.bd/, following the same process we used.*

Language and Presentation:

**Reviewer’s comments:**

The manuscript is written in clear and standard English. However, there are minor typographical and grammatical errors that should be addressed in the revision process. Enhancing the clarity of complex sections will also improve the manuscript's accessibility to a broader readership.

**Authors’ response:** We have cheeked the manuscript again and corrected typographical and grammatical errors and revised complex sentences to address your comment.

**Reviewer’s comments:**

Ethics and Data Protection: While the ethics section is present, more detail should be provided about participant consent, data protection measures, and the approval process by the ethics board. This will help meet international publication standards.

**Authors’ response:** We have updated the ethics approval section as follows to address your comment-

*The survey data we analyzed received ethical approval from the Ethics Committee of the Bangladesh Bureau of Statistics (BBS). Informed written consent was obtained from all participants or their legal guardians in cases where participants were minors (under 18 years old). We accessed the data from BBS by submitting a specific research proposal for this study. Before sharing the data, BBS de-identified it by removing all respondent-identifying variables. Since the data was de-identified, no further ethical approval was required.*
